# Using the value of Lin’s concordance correlation coefficient as a criterion for efficient estimation of areas of leaves of eelgrass from noisy digital images

**DOI:** 10.1186/s13029-014-0029-8

**Published:** 2014-12-20

**Authors:** Héctor Echavarría-Heras, Cecilia Leal-Ramírez, Enrique Villa-Diharce, Oscar Castillo

**Affiliations:** Centro de Investigación Científica y de Estudios Superiores de Ensenada, Carretera Ensenada-Tijuana No. 3918, Zona Playitas, Apdo. Postal 360, Código Postal 22860 Ensenada, B C México; Centro de Investigación en Matemáticas, A.C. Jalisco s/n, Mineral Valenciana, Guanajuato Gto., Código Postal 36240 México; Instituto Tecnológico de Tijuana, Tijuana, Baja California México

**Keywords:** Eelgrass leaf, Area estimations, Noisy digital images selection criterion, Concordance correlation coefficient

## Abstract

**Background:**

Eelgrass is a cosmopolitan seagrass species that provides important ecological services in coastal and near-shore environments. Despite its relevance, loss of eelgrass habitats is noted worldwide. Restoration by replanting plays an important role, and accurate measurements of the standing crop and productivity of transplants are important for evaluating restoration of the ecological functions of natural populations. Traditional assessments are destructive, and although they do not harm natural populations, in transplants the destruction of shoots might cause undesirable alterations. Non-destructive assessments of the aforementioned variables are obtained through allometric proxies expressed in terms of measurements of the lengths or areas of leaves. Digital imagery could produce measurements of leaf attributes without the removal of shoots, but sediment attachments, damage infringed by drag forces or humidity contents induce noise-effects, reducing precision. Available techniques for dealing with noise caused by humidity contents on leaves use the concepts of adjacency, vicinity, connectivity and tolerance of similarity between pixels. Selection of an interval of tolerance of similarity for efficient measurements requires extended computational routines with tied statistical inferences making concomitant tasks complicated and time consuming. The present approach proposes a simplified and cost-effective alternative, and also a general tool aimed to deal with any sort of noise modifying eelgrass leaves images. Moreover, this selection criterion relies only on a single statistics; the calculation of the maximum value of the Concordance Correlation Coefficient for reproducibility of observed areas of leaves through proxies obtained from digital images.

**Results:**

Available data reveals that the present method delivers simplified, consistent estimations of areas of eelgrass leaves taken from noisy digital images. Moreover, the proposed procedure is robust because both the optimal interval of tolerance of similarity and the reproducibility of observed leaf areas through digital image surrogates were independent of sample size.

**Conclusion:**

The present method provides simplified, unbiased and non-destructive measurements of eelgrass leaf area. These measurements, in conjunction with allometric methods, can predict the dynamics of eelgrass biomass and leaf growth through indirect techniques, reducing the destructive effect of sampling, fundamental to the evaluation of eelgrass restoration projects thereby contributing to the conservation of this important seagrass species.

## Background

Seagrass meadows are highly productive plant communities that grant valuable ecological services in estuaries and near-shore environments worldwide. Seagrasses provide food and shelter for a myriad of economically and ecologically valued marine organisms [1-3], play an important role in nutrient cycling [4,5], favor the stabilization of the shoreline as roots and rhizomes compact the substrate, preventing erosion [6,7], participate in the foundation of the detrital food web [8], and play also, a fundamental role in carbon sequestration [9]. Eelgrass (*Zostera marina L*.) is particularly relevant not only because it is the dominant seagrass species along the coasts of both the North Pacific and North Atlantic [10], but also, because eelgrass communities have been traditionally recognized as among the richest and most varied in the abundance of sea life [11]. Indeed, this cosmopolitan macrophyte was found to produce up to 64% of the total primary production of an estuarine system [12].

**Figure 1:**
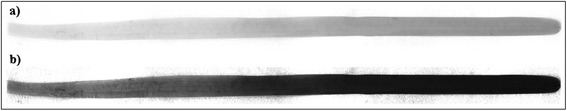
**A digital image of a** ***Zostera marina*** **leaf. a)** An image of *a Zostera marina* leaf exhibiting the typical belted shape. Related area is commonly approximated by the product of length and average width. **b)** The display of the image of the leaf using a darker tonality reveals pixels placed beyond the peripheral contours, which do not belong to the image and whose presence is explained by humidity- noise-related effects. Improper identification of the peripheral contour of the leaf image due to spurious entries can lead to miscalculation of related area.

The forcing of *Zostera marina* dynamics by environmental variables is well documented in the literature [13-18]. Light availability, temperature, and dissolved nutrients are the most important variables for explaining the observed variability [18,19]. But even when light and nutrients are not limiting, temperatures ranging above the upper limit tolerated by eelgrass can provoke severe negative effects on its growth [20]. Indeed, the onset of warm ENSO events has been shown to dramatically diminish eelgrass growth [20]. Therefore, the productivity of *Zostera marina* populations could be diminished by global climate change, which is expected to result in warming and rising seas, thereby reducing the availability of both light and nutrients underwater [21]. Another concern for the health of eelgrass populations pertains to increasing deleterious anthropogenic influences. The loss of eelgrass habitat has been noted worldwide, with major losses in the past few decades [22-25]. Within restoration strategies, replanting plays an important role [26-28]. The monitoring of these efforts is fundamental for the evaluation of the effectiveness of restoration of functions and values of natural populations. Accurate measurements of the standing crop and productivity of transplanted populations at a given time constitute an important input for evaluating the restoration of the ecological functions and values of natural populations. Although traditional assessment methods do not cause damage to natural populations, their invasive nature could significantly alter the development of transplanted populations. Echavarria-Heras et al. [29] and Echavarria-Heras et al. [30] propose allometric methods that reduce eelgrass biomass and leaf growth rate estimations to measurements of leaf length or area. Besides, the use of digital imagery could provide leaf area estimations which avoid invasive effects. But in some cases noise effects could lead to misidentification of pixels placed on the peripheral contour of leaves images (see Figure 1). This could spread uncertainty on leaf area estimations that ultimately could render imprecise allometric projections of biomass and leaf growth rates. Therefore, for accurateness we must rely on an image selection method that produces an unambiguous identification of the sequence of pixels that form the peripheral contours of digitalized eelgrass leaves. In order to achieve this task, there are techniques developed on the basis of the concepts of adjacency, vicinity, connectivity and tolerance of similarity between pixels (see Appendix). Using this framework Leal-Ramirez and Echavarria-Heras [31] introduced a direct comparison method aimed to discriminate the interval of tolerance of similarity that produces the most accurate estimations of length, width or area of eelgrass leaves from digital images with noise induced by humidity contents. For a given interval of tolerance of similarity, the process initially identifies the peripheral contour of the images of leaves and then measures the concomitant lengths widths and areas. Next, individual deviations between leaf area measurements taken from images and those obtained directly from leaves are used to produce statistics aimed to obtain the proportions of leaves for which image assessments underestimate or overestimate observed values. The ratio of these proportions defines a selection index whose smallest value provides criterion for choosing the interval of tolerance of similarity that yields the most accurate image related measurements. The implementation of the direct comparison method uses lengthy computational stages that include various statistical inferences on deviations between observed an image obtained leaf areas. In this contribution, we present an alternative criterion for the selection of the named interval of tolerance of similarity. The present procedure called the concordance correlation method; is simpler to implement than the direct comparison method. It only requires calculating the values of the Concordance Correlation Coefficient (CCC) for the reproducibility of observed leaf areas through proxies obtained from corresponding images. The present criterion proposes the use of the interval of tolerance of similarity that yields the maximum value of the aforementioned CCC for consistent digital image estimations of eelgrass leaves areas. Our results show that on spite of its simplicity the present selection criterion yields highly reliable levels of accuracy.

In section two, we present a brief review of the direct comparison method. Section three formally explains the present concordance correlation method. Section four describes the results of this study and discusses the advantages and possible drawbacks of the present approach.

### The Direct Comparison Method (DCM)

In this section we briefly describe the steps of the direct comparison method as conceived by Leal-Ramirez and Echavarria-Heras [31]. Initially, the DCM chooses a positive integer *n* and uses it to fix a tolerance level *q* = (*l*_*max*_/*n*), being *l*_*max*_ the maximum observed leaf length. This yields a covering for the range [0, *l*_*max*_] by a collection of *n* disjoint intervals of the form *I*_*k*_ = [*q*(*k* − 1), *qk*), with 1 ≤ *k* ≤ *n*. Subsequently, for each value of the index *k* the procedure identifies the group *G*_*k*_(*l*) of *n*_*k*_ leaves whose lengths are contained in *I*_*k*_. An index *j* such that 1 ≤ *j* ≤ *n*_*k*_ labels leaves in *G*_*k*_(*l*) while the symbols ${l}_{oj}^k$, ${h}_{oj}^k$ and ${a}_{oj}^k$ denote respectively the straight length, width and area of the *j*th leaf in *G*_*k*_(*l*). Particularly, estimations ${a}_{oj}^k$ of the leaf areas in *G*_*k*_(*l*) can be obtained by using the length times width proxy [32]. Digital images of leaves in the *G*_*k*_(*l*) groups are processed by a specified color format with a number *C*_*max*_ of colors and via intervals of tolerance of similarity *ST*(*r*) = [0, *r*], being *r*, 0 ≤ *r* ≤ *C*_*max*_ − 1, the number of different tonalities used for pixel identification. By keeping *ST*(*r*) fixed, a routine selects a starting point within the image of the *j*th leaf in *G*_*k*_(*l*) and detects all adjacent pixels falling within the selected interval of tolerance of similarity *ST*(*r*). This task which is achieved using equations (A1), (A2) and (A3) identifies the peripheral contour of the leaf image, and allows the measurements of the concomitant proxies for the length ${l}_{dj}^k(r)$, width ${h}_{dj}^k(r)$ and area ${a}_{dj}^k(r)$ of the leaf. Afterwards the method obtains the deviations for leaf length ${e}_{lj}^k(r)$, width ${e}_{hj}^k(r)$ and area ${e}_{aj}^k(r)$, given by: ${e}_{lj}^k(r)={l}_{oj}^k - {l}_{dj}^k(r)$, ${e}_{hj}^k={h}_{oj}^k-{h}_{dj}^k(r)$,and ${e}_{aj}^k(r)={a}_{oj}^k-{a}_{dj}^k(r)$. This produces respective average deviation values taken over groups *G*_*k*_(*l*). These are denoted by means of ${\overline{\delta}}_l^k(r)$, ${\overline{\delta}}_h^k(r)$, ${\overline{\delta}}_a^k(r)$, their corresponding averages taken over the whole collection of groups *G*_*k*_(*l*) by means of ${\overline{\delta}}_l(r)$, ${\overline{\delta}}_h(r)$, ${\overline{\delta}}_a(r)$ and the associated standard deviations through *σ*_*δl*_(*r*), *σ*_*δh*_(*r*) and *σ*_*al*_(*r*) respectively. Then, for each range of similarity *ST*(*r*), the technique identifies the leaves satisfying the conditions

1 $$ {\overline{\delta}}_h(r)\ge 0, $$

2 $$ {\overline{\delta}}_l(r)\ge 0, $$

3 $$ {\overline{\delta}}_l(r)-{\sigma}_{\delta l}(r)\le {\overline{\delta}}_l^k(r)\le {\overline{\delta}}_l(r)+{\sigma}_{\delta l\kern0.5em }(r), $$

4 $$ {\overline{\delta}}_h(r)-{\sigma}_{\delta h}(r)\le {\overline{\delta}}_h(r)\le {\overline{\delta}}_h(r)+{\sigma}_{\delta h}(r) $$

and

5 $$ {e}_{aj}^k(r)\ge 0 $$

and use their area values ${a}_{dj}^k(r)$ to calculate *λ*_*a*_(*r*), which stands for the proportion of images of leaves for which *a*_*d*_ produces consistent estimations of observed leaf areas *a*_0_. This proportion is calculated according to the formula,

6 $$ {\lambda}_a(r)=\frac{{\displaystyle {\sum}_{k=1}^n}{\displaystyle {\sum}_{j=1}^{n_k}}\left[{a}_{dj}^k(r)\ \Big|\ \mathrm{leaves}\ \mathrm{in}\ {G}_k(l)\ \mathrm{that}\ \mathrm{comply}\ \mathrm{with}\ \mathrm{conditions}\ (1)\mathrm{through}(5)\ \right]}{{\displaystyle {\sum}_{k=1}^n}{\displaystyle {\sum}_{j=1}^{n_k}}{a}_{oj}^k} $$

Then the method obtains the proportion *β*_*a*_(*r*) of images of leaves for which *a*_*d*_ estimations overestimates observed leaf areas *a*_*o*_ which is calculated through *β*_*a*_(*r*) = 1 − *λ*_*a*_(*r*), and use *λ*_*a*_(*r*) and *β*_*a*_(*r*) to calculate the value of the image selection index *IS*(*r*), formally defined by

7 $$ IS(r) = {\beta}_a(r)\kern0.10em /\kern0.15em {\lambda}_a(r) $$

Finally, the DCM proposes the use of the *ST*(*r*) interval producing the smallest value of *IS*(*r*) for reliable estimation of the areas of leaves of eelgrass using images whose peripheral contour is distorted by noise induced by humidity contents.

### The Concordance Correlation Method (CCM)

The Concordance Correlation Coefficient symbolized by mean of $\rho$ [33,34] is used to determine reproducibility, as it measures the agreement between the variables *x* and *y* by appraising the extent to which they fall on the 45° line through the origin. Its numerical value is represented in terms of the ratio of the expected orthogonal squared distance from the diagonal *y* = *x* to the expected orthogonal squared distance from the diagonal *y* = *x* assuming independency. The value of $\rho$, is commonly used to assess how well a new set of observations *y* reproduce an original set *x*. When $\rho$ is computed on a $m$-length data set (i.e., two vectors (*x*_1_, *x*_2_, ⋯, *x*_*m*_) and (*y*_1_, *y*_2_, ⋯, *y*_*m*_) the resulting statistics is denoted by means of $\widehat{\rho}$ and calculated through

8 $$ \widehat{\rho}=\frac{2{s}_{xy}}{s_x^2+{s}_y^2 + {\left(\overline{x}+\overline{y}\right)}^2}, $$

being

9 $$ \overline{x}=\frac{1}{m}{\displaystyle \sum_{j=1}^m}{x}_j $$

10 $$ {s}_x^2=\frac{1}{m}{\displaystyle \sum_{j=1}^m}\ {\left({x}_j-\overline{x}\right)}^2 $$

and

11 $$ {s}_{xy}=\frac{1}{m}{\displaystyle \sum_{j=1}^m}\left({x}_j-\overline{x}\right)\left({y}_j-\overline{y}\right) $$

In the present work the value of $\widehat{\rho}$ will provide a criterion for the incumbent digital image selection process. The linked CCM does not require the sorting of observed leaf lengths into the *G*_*k*_(*l*) groups of the DCM. As it is done in the DCM, in the present CCM, the digital images of sampled leaves are primarily processed by a specified color format with a number *C*_*max*_ of colors and using intervals of tolerance of similarity *ST* (*r*) = [0, *r*] with 0 ≤ *r* ≤ *C*_*max*_ − 1. Again by keeping *ST*(*r*) fixed and within the *jth* leaf image, a routine selects a starting point, and using Eqs. (A1), (A2) and (A3) detects all adjacent pixels connected within the realm of the designated interval of tolerance of similarity *ST*(*r*). This device identifies the peripheral contour of the leaf image allowing associated measurements of length *l*_*dj*_(*r*) and width *h*_*dj*_(*r*) whose product for 1 ≤ *j* ≤ *m*, yields image estimated leaf areas *a*_*dj*_(*r*). Instead of performing the statistical steps required to calculate *IS*(*r*), simply for *r* fixed in equations (9), (10) and (11) we make *x*_*j*_ stand for observed leaf area measurements (*a*_01_, *a*_02_, ⋯, *a*_0*m*_) and let *y* match digital image produced estimations (*a*_*d*0_(*r*), *a*_*d*1_(*r*), ⋯, *a*_*dm*_(*r*)). Then equation (8) yields the resulting value of the Concordance Correlation Coefficient. In the present settings this will be denoted through by means of the symbol $\widehat{\rho}(r)$ to emphasize its dependence on *r*, that is, changing *ST*(*r*) produces different pairs of observed and image calculated leaf areas (*a*_0*j*_ , *a*_*dj*_(*r*)), 1 ≤ *j* ≤ *m*, as well as different values of the associated $\widehat{\rho}(r)$. After all values of *r* in the chosen color format are exhausted, we select the tolerance of similarity interval *ST*(*r*) that produces the highest value for $\widehat{\rho}(r)$ for efficient estimation of eelgrass leaves area from digital images with noise related to environmental factors.

## Results and discussion

For the purposes of the present study, we used a data set obtained by randomly sampling 5 shoots biweekly from January through December 2009 in a *Zostera marina* field at Punta Banda estuary, a shallow coastal lagoon located near Ensenada, Baja California, Mexico (31° 43–46 N and 116° 37–40 W). For each sampled leaf, a millimeter ruler was used to obtain leaf length measurements *l*_*o*_ to the nearest 1/10 *mm* taken as the distance from the top of the sheath to the leaf tip. Meanwhile, observed leaf width *h*_*o*_ was measured at a point halfway between the top of the sheath and the tip [32]. Observed leaf area estimations *a*_*o*_ were calculated by means of length times width proxy *a*_*o*_ = *l*_*o*_ ⋅ *h*_*o*_.

We obtained *l*_*max*_ = 460 *mm*. For the data grouping required by the DCM we choose *n* = 46 so we acquired *q* = 10 *mm*, and for the interval [0, *l*_*max*_] we formed a partition ${P}_0^{460}$ of disjoint intervals *I*_*k*_ of the form *I*_*k*_ = {*l* | *q*(*k* − 1) ≤ *l* < *qk*}, with 1 ≤ *k* ≤ 46. Hence, for each value of the index *k*, we formed a group *G*_*k*_(*l*) containing leaves with sizes varying in the interval *I*_*k*_. Longer and older leaves displayed darker tonalities than younger and shorter ones, but leaves with lengths varying on a given partition interval *I*_*k*_ displayed a similar color distribution. For some of the partition intervals there was at most one leaf with length placed in the linked variation range. Therefore, these groups are not taken into account because they do not provide information for the statistical analysis.

**Figure 2:**
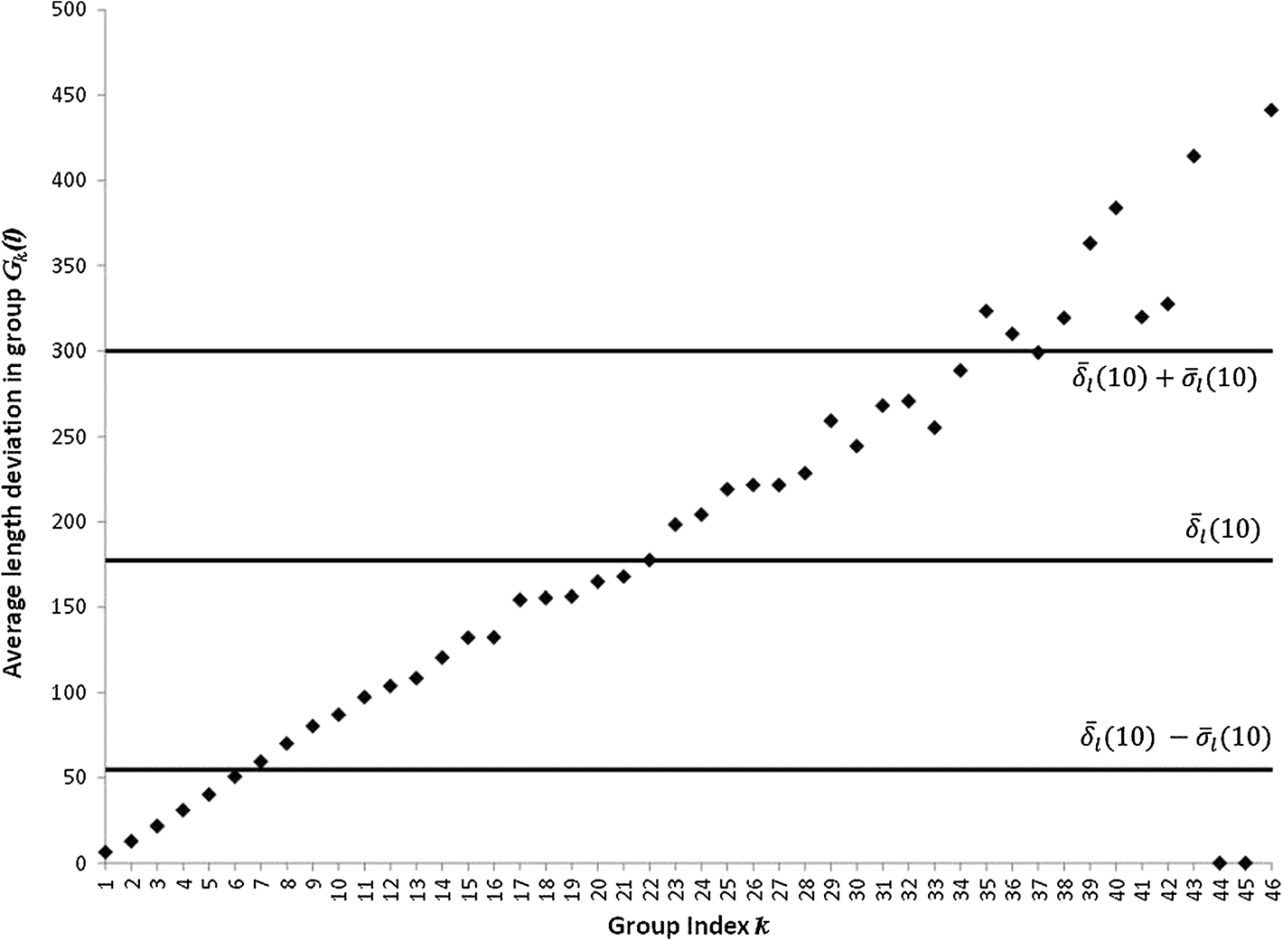
**The effect of similarity index** ***r*** **= 10 on average deviations** ${\overline{\boldsymbol{\delta}}}_{\boldsymbol{l}}^{\boldsymbol{k}}\left(\boldsymbol{r}\right)$ **.** For *r* = 10 a regular tendency of ${\overline{\delta}}_l^k(r)$ depending on group index *k* is shown. This yields a large proportion of groups with ${\overline{\delta}}_l^k(r)$ lying outside the interval bounded by ${\overline{\delta}}_l(r)+{\sigma}_{\delta l}(r)$ and ${\overline{\delta}}_l(r)-{\sigma}_{\delta l}(r)$ (cf. inequality 3).

**Figure 3:**
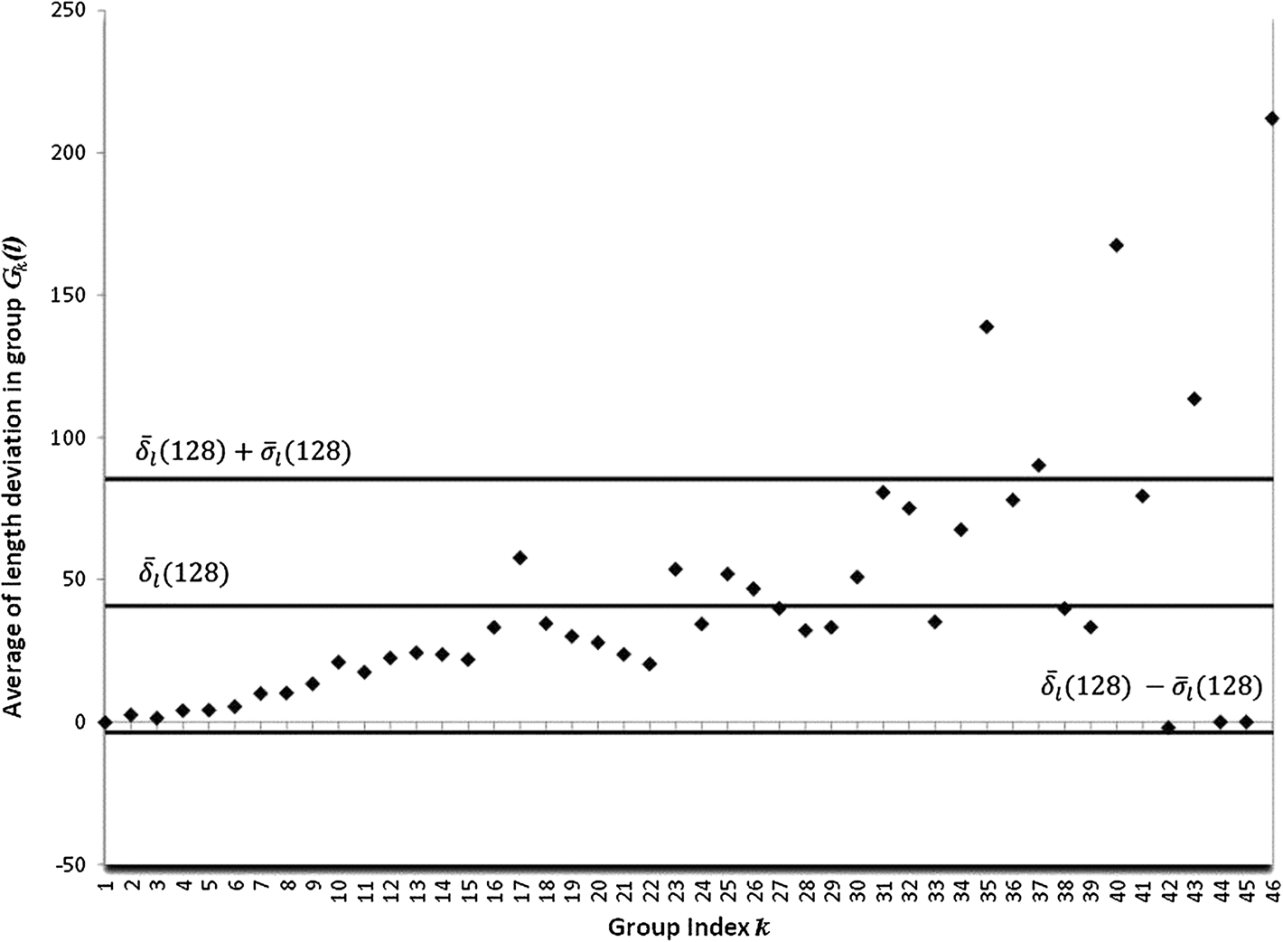
**The effect of similarity index** ***r*** **= 128 on average deviations** ${\overline{\boldsymbol{\delta}}}_{\boldsymbol{l}}^{\boldsymbol{k}}\left(\boldsymbol{r}\right)$ **.** For *r* = 128 the regular tendency of increasing ${\overline{\delta}}_l^k(r)$ values shown figure 2 is no longer observed and a reduced number of ${\overline{\delta}}_l^k(r)$ values lying outside the interval bounded by ${\overline{\delta}}_l(r)+{\sigma}_{\delta l}(r)$ and ${\overline{\delta}}_l(r)-{\sigma}_{\delta l}\left(\operatorname{r}\right)$ is observed.

**Figure 4:**
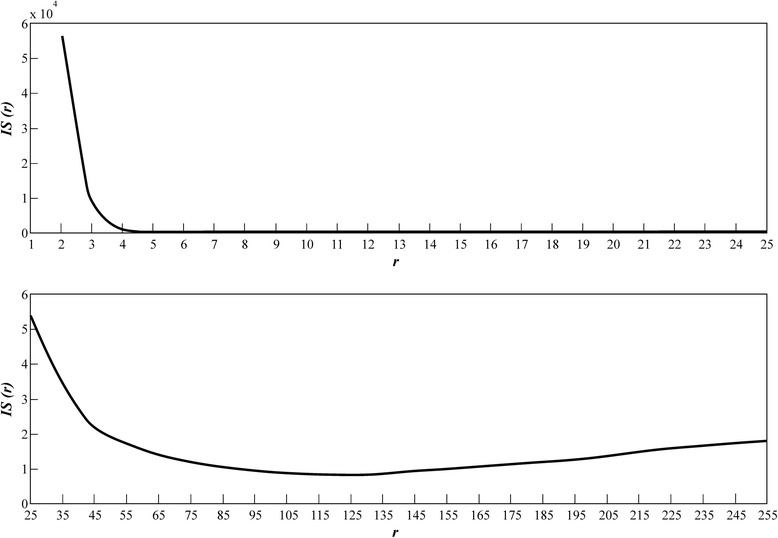
**The behavior of the** ***IS*** ***(r)*** **selection index through the interval 1 ≤** ***r*** **< 255.** For small values of *r* the interval of tolerance of similarity *ST*(*r*) does not include the necessary tonalities that the image identification procedure requires. Therefore identification of pixels within an image can be expected to be imprecise. Consequently reduced values of *λ*_*a*_(*r*), will be expected, which lead to large values of the *IS*(*r*) selection index. For *r* ≥ 25, values of *IS*(*r*) decrease until its minimum value is attained at *r* = 128. According to the DCM selection criterion for the present data, both an RGB color format and *ST*(128) interval of tolerance of similarity can be used for efficient estimation of areas of eelgrass leaves using images with noise induced by humidity contents.

According to the DCM, for each leaf belonging to the group *G*_*k*_(*l*) we obtained its digital image. For dealing out with all these individual images we selected an RGB color format with a number *C*_*max*_ of 256 colors. For processing each one of the available leaves images, we choose different tolerance of similarity levels *ST*(*r*) = [0, *r*] with the upper bound *r* satisfying 0 ≤ *r* ≤ *C*_*max*_ − 1. Then for a given *ST*(*r*) range, we selected a starting point inside the considered leaf image, and identified using equations (A1), (A2) and (A3) all adjacent pixels falling within the named similarity range *ST*(*r*). This recognizes the outer contour of the digital blade, and produce concomitant leaf width, length and area estimations. The next step in the DCM concerns the calculation of the selection index *IS*(*r*) which depends on the value of the *λ*_*a*_(*r*) statistics. But according to equation (6) obtaining the value of *λ*_*a*_(*r*) requires counting the number of leaves in each group *G*_*k*_(*l*), that comply with conditions (1) through (5) and these numbers depend on the chosen value of *r*. Moreover, for small values of *r* the number of different tonalities included in *ST*(*r*) is limited so identification of pixels within an image can be expected to be imprecise. This is handily clarified by Figure 2, produced using *r* = 10 and that shows a systematic tendency for average length deviations ${\overline{\delta}}_l^k(r)$ depending on group index *k.* As a result we can observe a large number of ${\overline{\delta}}_l^k(r)$ values lying above the ${\overline{\delta}}_l(r)+{\sigma}_{\delta l}(r)$ and beyond the ${\overline{\delta}}_l(r)-{\sigma}_{\delta l}(r)$ thresholds in inequality (3). The bigger the value of *r*, the greater the number of color tonalities included in the interval *ST*(*r*) and precision in image contour identification improves. This is observed in Figure 3, produced using *r* = 128 and which does not display the above quoted systematic tendency, but a reduced number of groups of leaves with average length deviations ${\overline{\delta}}_l^k(r)$ lying outside the interval bounded by ${\overline{\delta}}_l(r)+{\sigma}_{\delta l}(r)$ and ${\overline{\delta}}_l(r)-{\sigma}_{\delta l}(r)$. Consequently, for small values of *r* we can expect reduced values of *λ*_*a*_(*r*) and as a result according to equation (7) large values of *IS*(*r*). Additionally, when the interval of tolerance of similarity becomes wider, smaller values of *IS*(*r*) can be expected. In fact, as shown in Figure 4, the DCM captures this effect in a consistent way, with small values of *r* leading to large values for the selection index *IS*(*r*). Moreover, through the interval 1 ≤ *r* < 128, *IS*(*r*), decreases reaching a minimum value of 0.91, attained at *r* = 128. Meanwhile, for *r* ≥ 128, the values of the selection index *IS*(*r*) steadily increased towards a value of 1.84, attained at *r* = 255. Therefore, according to the DCM selection criterion *ST*(128) must be chosen for efficient estimation of areas of eelgrass leaves using images with noise induced by humidity contents.

**Figure 5:**
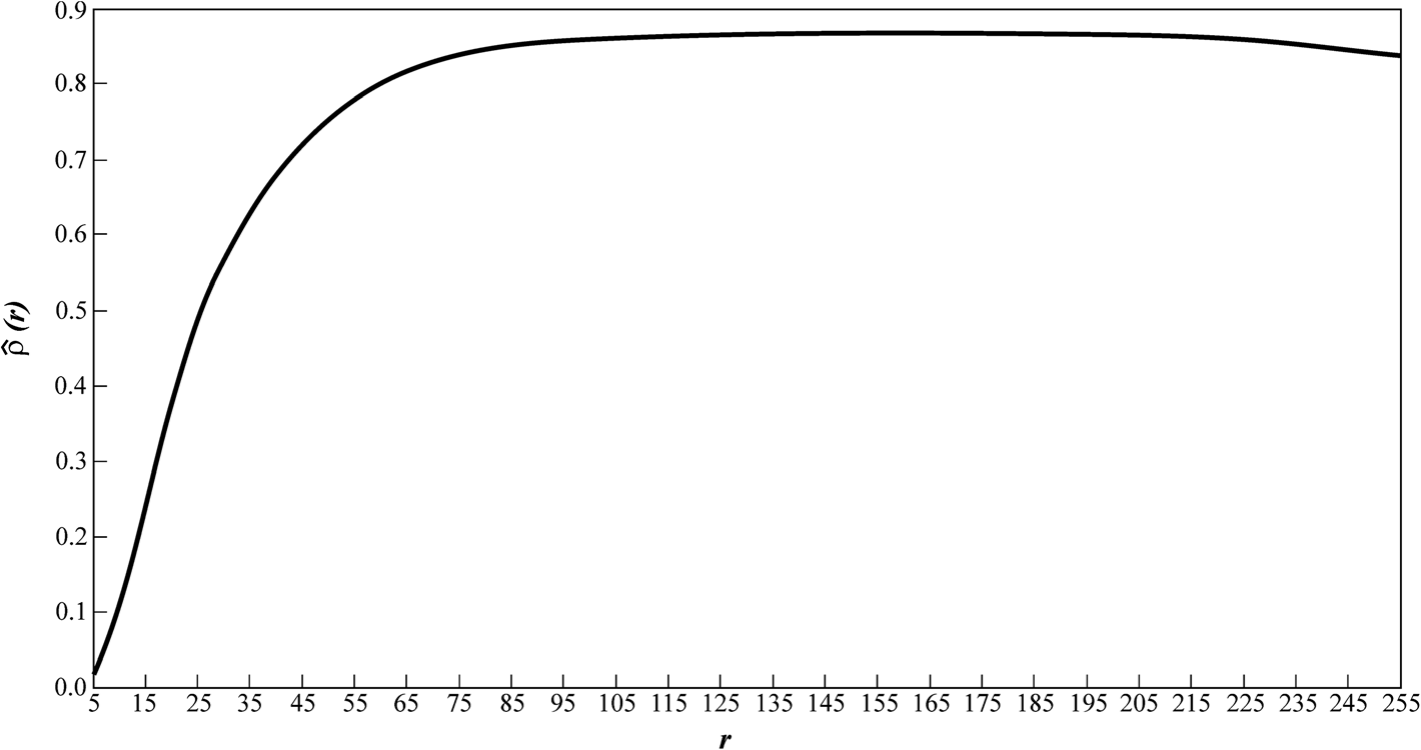
**The behavior of the concordance correlation coefficient** $\widehat{\boldsymbol{\uprho}}\left(\boldsymbol{r}\right)$ **, through the interval 1 **≤ ***r*** **< 255.** Increasing values of $\widehat{\rho}(r)$ through the interval 1 ≤ *r* < 128 are displayed. This means that the wider the interval of similarity *ST*(*r*) the greater the reproducibility of observed leaf areas by image proxies becomes. Interestingly through the domain 128 ≤ *r* < 178 values of $\widehat{\rho}(r)$ are maintained within a plateau of slight variation around $\widehat{\rho}\left(128\ \right)=0.90$. Afterwards, for 178 ≤ *r* ≤ 255 values of $\widehat{\rho}(r)$ decrease slightly until $\widehat{\rho}(r)$ drops to a value of 0.8464 attained at *r* = 255. Then for values of *r* larger than *r* = 128 reproducibility is not improved and coinciding with the criterion in the DCM for the present data, both an RGB color format and the *ST*(128) interval of tolerance of similarity could be used for image selection when noise due to humidity contents is present and efficient estimations of eelgrass leaf area taken from these images is required.

Now for the CCM, since a small value of *r* fails to recognize some pixels in the digital image, we might expect a low reproducibility of directly obtained measurements (*a*_01_, *a*_02_, ⋯, *a*_0*m*_) by means of digitally obtained proxies (*a*_*d*0_(*r*), *a*_*d*1_(*r*), ⋯, *a*_*dm*_(*r*)). This is indeed shown in Figure 5. Moreover , the larger the value of *r*, the greater the number of color tonalities included in the interval *ST*(*r*), as a result exactness in image contour identification increases, and reproducibility improves, this explaining why Figure 5 shows increasing values of $\widehat{\rho}(r)$ through the interval 1 ≤ *r* < 128. Moreover, through the domain 128 ≤ *r* < 178 the values of $\widehat{\rho}(r)$ are maintained within a plateau of slight variation around $\widehat{\rho}(128) = 0.90$, but for 178 ≤ *r* ≤ 255, $\widehat{\rho}(r)$ decreases dropping to a value of 0.8464, attained at *r* = 255. Thenceforth, intervals of tolerance of similarity, wider than *ST*(128) do not improve reproducibility of observed values of leaves areas by means of their image obtained surrogates. Thus, for the sake of accuracy and simplicity, *ST*(128) should be used for image selection when noise due to environmental factors is present and efficient estimations of eelgrass leaf area taken from these images are required.

**Figure 6:**
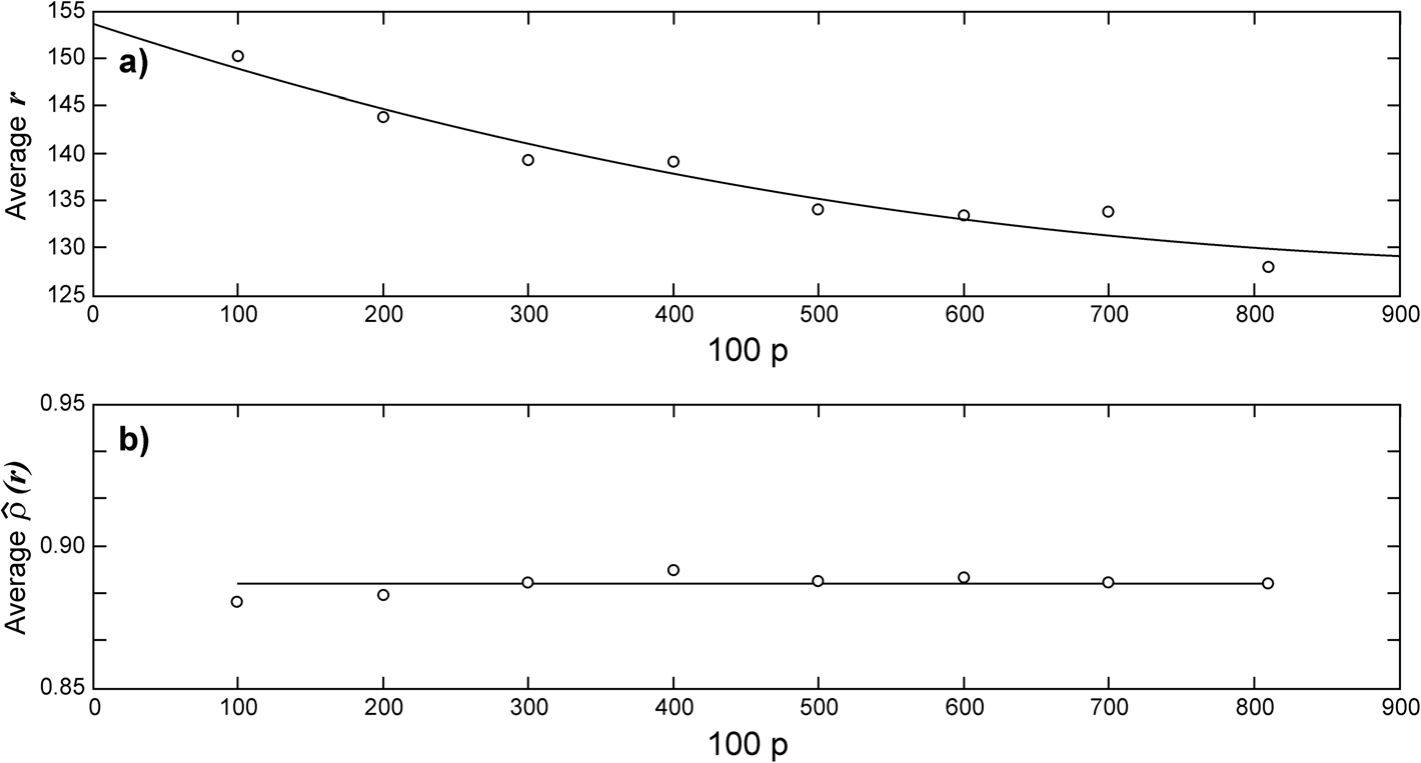
**Dependence of both the value of** ***r*** **for maximum** $\widehat{\boldsymbol{\rho}}\left(\boldsymbol{r}\right)$ **and the maximum value of** $\widehat{\boldsymbol{\rho}}\left(\boldsymbol{r}\right)$ **itself on sample size.** For each value of the sample size index *p* = 1, 2, … 8 a fixed number *s*(*p*) of samples of size 100*p* each were uniformly drawn from the population of observed leaf areas. For each one of the samples in a set *s*(*p*), we iterated values of *r* through the interval 1 ≤ *r* ≤ 255, and for each one of these *r* values we obtained the concomitant concordance correlation coefficient values $\widehat{\rho}(r)$. We repeated this procedure for all the samples in the set *s*(*p*) and averaged the *r* values at which $\widehat{\rho}(r)$ attained its maximum value, the obtained averages for the different values of the sample size index *p* are shown in **a)**. The maximum values that $\widehat{\rho}(r)$ obtained in a sample were also averaged over the *s*(*p*) sets. These average values depending on sample size are correspondingly shown in **b)**. The results of this study shows that neither the optimal interval of tolerance of similarity *ST*(*r*) or the reproducibility of observed leaf areas by means of their digital image surrogates depend on sample size, therefore the CCM can be considered as, a robust procedure.

In order to assess robustness of the CCM, we performed a resampling experiment. We chose a sample size index *p* = 1, 2, …, 8 then for each value of *p* a set *s*(*p*) of samples of size 100*p* each were uniformly drawn from the (*a*_01_, *a*_02_, ⋯, *a*_0*m*_) population. Next, we selected one of the *s*(*p*) samples, for each value of *r* through the interval 1 ≤ *r* ≤ 255, we designated the matching areas obtained from digital images and calculated the concomitant Concordance Correlation Coefficient values $\widehat{\rho}(r)$. We recorded the value of *r* at which the maximum for $\widehat{\rho}(r)$ was attained for the selected sample. We repeated this procedure for all the samples in the set *s*(*p*) and then averaged the obtained *r* values for maximum $\widehat{\rho}(r)$. Figure 6 displays the obtained averages for the different values of the sample size index *p*. The maximum values of $\widehat{\rho}(r)$ per sample were also averaged over the *s*(*p*) sets. These last average values are shown in Figure 6. The results of this study show that the optimal interval of tolerance of similarity, as well as, the reproducibility of observed leaf areas by means of their digital image surrogates can be considered independent of sample size. Therefore, the CCM can be regarded as a robust procedure.

According to our results, both methods sustain the same conclusion regarding the choosing of *ST*(128) on behalf of accuracy. However, in comparison to the complicated multi-stage procedures of the DCM, using $\widehat{\rho}(r)$ values provide a direct and simpler criterion for choosing an interval of tolerance of similarity *ST*(*r*) for reliable digital image related assessments of eelgrass leaf area under the specified noise effects. But the main advantage of the CCM resides on the fact that it allows a straightforward interpretation of the addressed digital image selection procedures in terms of a measure of reproducibility. Indeed the plateau in $\widehat{\rho}(r)$ values linked to the domain 128 ≤ *r* ≤ 178, and the subsequent decreasing mode associated to *r* ≥ 178 shown in Figure 5 indicate that intervals of tolerance of similarity wider than *ST*( 128) will fail to improve reproducibility of observed values of leaf area by means of their image produced proxies. In other words for *r* ≥ 128, *ST*(*r*) includes more tonalities than those contained within the real image, thereby favoring the incorporation of spurious entries appearing beyond its peripheral contour and within the framing of the image. Thus including more color tonalities than necessary in the image processing task could not grant a gain in accuracy, but instead, depending on the severity of the noise effects (Figure 1), and on the size of the framing enclosing the peripheral contour of the image (Figure 1), more spurious pixels could be taken in to account by the image processing devise, which could lead to increased miscalculation of leaf area obtained from images. Meanwhile, our analysis confirms that when noise induced into images by the humidity contents of the leaves reduces the accuracy of estimations of the associated areas we could use a RGB color format, an *ST*( 128) interval of tolerance of similarity and equations (A1), (A2) and (A3) to identify the peripheral contour of leaves images for optimal reproducibility.

## Conclusions

The results of the present digital image selection procedure provide simple, unbiased and non-destructive measurements of eelgrass leaf area. These measurements in conjunction with allometric methods [35] can predict the dynamics of biomass and leaf growth through indirect techniques, reducing the destructive effect of sampling and simplifying time consuming methods in the laboratory [36]. Nevertheless, it is worth to emphasize, that leaves removed from a shoot readily begin to lose water and degrade, so changes in shape may occur [37]. Therefore, even though humidity contents could certainly induce noise effects, an efficient digitalizing of a *Zostera marina* blade requires the maintenance of an optimal humidity for increased image fidelity. By taking this into account we can assert that the apparent similarity of values of $\widehat{\rho}(r)$ linked to the interval 128 ≤ *r* ≤ 178 could not be exhibited as a weakness of the CCM, that is, the plateau shown in Figure 5 does not associate to vagueness in the imbedded selection criteria. Indeed in this study both the preparation of lives before digitalization procedures and the framing used to bound the area surrounding the peripheral contour of the digital leaves was effective (1) for reducing inconsistencies attributable to a biased mapping of leaf shape into images, (2) by lessening bias due to the inclusion of spurious entries linked to noise into images and (3) because the framing size used in the present identification procedure further limited the participation of spurious entries in image processing tasks. Therefore, *r* = 128 (that is, the entrance threshold for the plateau of maximum $\widehat{\rho}(r)$ values in Figure 5) includes the required number of different tonalities for the processing of the present set of images and we choose it for a consistent estimation of the pertinent leaf area. Although, in the present settings the aforementioned bias reduction practices explain why values of *r* beyond *r* = 128 sustain the same selection criterion, using *r* ≥ 128 could lead to extended time consuming computational procedures, because more than necessary tonalities will be included in the identification undertaking. It is also worth to highlight that in further applications, before the CCM could provide consistent results, care should be taken in order to ensure that the handling of samples be performed in an efficient way for reducing bias in the overall image selection procedures. Indeed we could anticipate that in settings where points (1) through (3) above are disregarded, the inherent bias could seriously reduce reproducibility. Nevertheless, this could not be exhibited as a weakness of the present CCM, since the DCM itself as well as any other image selection procedure is subject to the same bias effects. In summary, the CCM, not only provides a simplified and robust image processing device, besides, (a) this criterion offers a conceptual substantiation for the DCM itself by linking the minimum values of the selection index *IS*(*x*), to the maximum values of the Concordance Correlation Coefficient $\widehat{\rho}(r)$, and (b) even though here we applied the CCM to account solely for the effects of noise linked to humidity contents, it is worth to mention that since the core of the CCM criterion is the evaluation of reproducibility, its scope directly embraces the treatment of any kind of noise effects that can reduce the accurateness of digital image proxies of areas of eelgrass leaves.

Studies of seagrass communities such as those composed of *Zostera marina* show that these systems are among the most productive marine systems [38]. The characterization of the dynamics of such ecosystems is important from both a scientific and conservation perspective. Moreover, the methods sustained by the present research may be fundamental to the evaluation of eelgrass restoration projects and could thereby contribute to the conservation of this important seagrass species.

## Appendix

We describe here the conceptual and formal framework for digital image processing. Two pixels are adjacent if, and only if, they share one of their borders, or at least one of their corners. Two pixels are neighbors if they fulfill the definition of adjacency. Formally, the vicinity *V*_*p*_(*x*, *y*) of the point *P*(*x*, *y*) is defined through

A1 $$ {V}_p\left(x,y\right)=\left\{\begin{array}{c}\hfill \left(x+1,y\right),\left(x-1,y\right),\left(x,y+1\right),\left(x,y-1\right),\hfill \\ {}\hfill \left(x+1,y+1\right),\left(x+1,y-1\right),\left(x-1,y+1\right),\left(x-1,y-1\right)\hfill \end{array}\right\} $$

Without loss of generality, we explain the notion of tolerance of similarity, by referring to the Reed, Green and Blue (RGB) color space. This allows quantifying tonality in terms of the intensities of the constituting primary colors: red, green, and blue. To indicate at which amount each one of these colors is mixed, to produce a given tonality a value is assigned to each prime color, for example, the value 0 means that a given primary color does not appear in the mix, but if a chief color component is non-vanishing it means that it contributes to the mix in a given intensity. We introduce *C*_*max*_ which identifies the number of colors to be used through the whole image processing task. For an RGB color space we have *C*_*max*_ = 256. Usually, the intensity of each of the primary colors appearing in a mix is measured on a scale ranging from 0 to *C*_*max*_ − 1. The set of all color intensities can be represented in the form of a cube in the Cartesian coordinate system, where each color is a point on the surface or in its interior. Given points *P* = (*p*_1_, *p*_2_, …, *p*_*n*_) and *Q* = (*q*_1_, *q*_2_, …, *q*_*n*_) in an RGB color space, we will define the distance *d*_*E*_(*P*, *Q*) between them through,

A2 $$ {d}_E\left(P,Q\right) = \sqrt{{\displaystyle \sum_{i=1}^n}{\left({p}_n-{q}_n\right)}^2} $$

Moreover, given a point *P* in an RGB color space, a second one *Q* with the greatest similarity to *P* is the one placed at the smallest distance *d*_*E*_(*P*, *Q*). Furthermore, let *ST*(*r*) = [0, *r*] be a color tonality range, being *r* the number of different colors included. Then, we must have 1 ≤ *r* ≤ *C*_*max*_ − 1 and we will say that two pixels *P* and *Q* are similar to a tolerance limit *ST*(*r*) if the inequality

A3 $$ {d}_E\left(P,Q\right)\le r $$

is satisfied. The range *ST*(*r*) is called “interval of tolerance of similarity” and the upper bound *r* can be interpreted as the maximum distance that two points located within the extent of an object can attain in a RGB color space in order to be considered similar. Connectivity between pixels is used to identify the limits in objects and regions in an image. We will say that two pixels *P* and *Q* are connected with tolerance of similarity *ST*(*r*) if they fulfill the definition of adjacency and also if inequality (A3) holds.
